# Sociodemographic and genetic determinants of nonalcoholic fatty liver disease in type 2 diabetes mellitus patients

**DOI:** 10.1186/s43141-022-00349-w

**Published:** 2022-04-29

**Authors:** Muhammad Adnan, Abdul Wajid, Wasif Noor, Andleeb Batool, Muhammad Aasim, Kamran Abbas, Quratul Ain

**Affiliations:** 1 Health Research Institute, National Institute of Health, Lahore, Pakistan; 2 Department of Molecular Biology, Virtual University of Pakistan, Lahore, Pakistan; 3 Diabetes Clinic, Sir Ganga Ram Hospital, Lahore, Pakistan; 4 Department of Zoology, Government College University, Lahore, Pakistan

**Keywords:** Diabetes mellitus type 2, Nonalcoholic fatty liver disease, Obesity, Polymorphism genetic, Risk factors

## Abstract

**Background:**

Nonalcoholic fatty liver disease (NAFLD) showed significant association with *PNPLA3* rs738409 polymorphism in unrelated individuals. However, it is still unknown whether the relationship of NAFLD with *PNPLA3* variant exists or not among subjects with type 2 diabetes mellitus (T2DM). Therefore, the study aimed to evaluate sociodemographic and genetic determinants of NAFLD in type 2 diabetics.

**Methods:**

The cross-sectional analytical study was conducted at the Department of Molecular Biology, Virtual University of Pakistan, Lahore, Pakistan, during 2019–2020. A total of 153 known cases of T2DM were enrolled using convenience sampling. After excluding patients (*n* = 24) with HCV, alcoholism, or missing information, data from 129 eligible diabetics with and without NAFLD were analyzed using SPSS. Odds ratios using crosstabs and adjusted odds ratios using binary and multinomial logistic regression were calculated to measure the risk of NAFLD.

**Results:**

Adults 18–35 years were 7.0%, 36–55 years were 64.3%, ≥ 56 years were 28.7%, and females were 66.7%. A total of 41.1% of patients had obesity, 52.7% had NAFLD, and 29.05% carried mutant G allele of rs738409 polymorphism. Among overall diabetics, NAFLD showed association with female (*OR* = 2.998, *p* = 0.007), illiterate (*OR* = 3.067, *p* = 0.005), and obese (*OR* = 2.211, *p* = 0.046) but not with *PNPLA3* genotype under any model (all *p* = > 0.05). Among obese diabetics, NAFLD showed association with female (*AOR* = 4.010, *p* = 0.029), illiterate (*AOR* = 3.506, *p* = 0.037), GG + CG/CC (*AOR* = 3.303, *p* = 0.033), and GG/CG + CC (*AOR* = 4.547, *p* = 0.034) using binary regression and with female (*AOR* = 3.411, *p* = 0.051), illiterate (*AOR* = 3.323, *p* = 0.048), GG + CG/CC (*AOR* = 3.270, *p* = 0.029), and GG/CG + CC (*AOR* = 4.534, *p* = 0.024) using multinomial regression.

**Conclusions:**

NAFLD and obesity were the most common comorbid diseases of T2DM. Gender female, being illiterate, and *PNPLA3* rs738409 polymorphism were significant risk factors of NAFLD among obese diabetic patients.

## Background

Nonalcoholic fatty liver disease (NAFLD) is a polygenic and heritable disorder [1], characterized by excess accumulation of fat in the liver parenchyma without history of alcoholism and hepatitis [2]. Its prevalence is 25.0% in the general adult population [3] and 50.0 to 70.0% in patients with type 2 diabetes mellitus (T2DM) [4]. NAFLD increases the risk of developing T2DM, whereas diabetes increases the progression of NAFLD to nonalcoholic steatohepatitis (NASH) and the risk of cirrhosis and hepatocellular carcinoma (HCC). Hence, a two-way relationship is present between NAFLD and T2DM [5].

Various demographic and genetic factors demonstrated greater risk for NAFLD. Age [6, 7], gender [6–8], ethnicity [9], metabolic syndrome (MetS), and its components including dyslipidemia, obesity, hypertension (HTN), and T2DM [10] were associated with the risk of NAFLD. Genetic factors such as the patatin-like phospholipase domain containing 3 (*PNPLA3*), the transmembrane 6 superfamily member 2 (*TM6SF2*), the membrane-bound *O*-acyltransferase domain containing 7 (*MBOAT7*), and the glucokinase regulator (*GCKR*) [11] were also associated with the risk of NAFLD. The product of human *PNPLA3* gene, i.e., triacylglycerol lipase enzyme mediates hydrolysis of triacylglycerol (TAG) in adipocytes. However, the substitution of isoleucine with methionine at position 148 (I148M) causes a loss of function [12]. Genome-wide association studies (GWAS) identified several genes such as the glucokinase (*GCK*), the glucokinase regulator (*GCKR*), the transcription factor 7-like 2 (*TCF7L2*), the hepatocyte nuclear factor-1A (*HNF1A*), and fat mass and obesity-associated (*FTO*) gene playing a role in developing T2DM [13]. In people with T2DM, *PNPLA3* I148M or rs738409 polymorphism showed significant association with liver fibrosis independent of body mass index (BMI) [14] and with the risk of increased liver fat content (LFC) independent of serum lipids [15], but not with susceptibility of NAFLD [16]. However, it revealed association with the risk and severity of NAFLD in meta-analyses of case-control studies on unrelated individuals [17–19]. The variations across studies in terms of characteristics of study population, selection of controls, laboratory methods, and statistical approaches lead to the ambiguity, whether or not these demographic and genetic factors, particularly rs738409 polymorphism, are associated with NAFLD in T2DM cases. Therefore, the present study aimed to determine the sociodemographic and genetic determinants of NAFLD in T2DM patients.

## Methods

### Ethical approval

The study was approved by the subcommittee of Advanced Studies and Research Board (ASRB) of the Faculty of Science and Technology, Virtual University of Pakistan, Lahore, Pakistan, vide letter no.VU/ASRB/214-5 dated December 02, 2019. Written informed consent was sought from all volunteer patients.

### Design, setting, and duration of study

The cross-sectional analytical study was conducted at the Department of Molecular Biology, Virtual University of Pakistan, Lahore, Pakistan, during 2019–2020.

### Characteristics, size, and selection of sample

A total of 153 known T2DM patients, of age 18–90 years, both male and female patients, belonging to any income group, caste or area of Pakistan, were enrolled by non-probability convenience sampling technique. None of 153 patients was reactive to hepatitis B surface antigen (HBsAg); however, patients reactive to anti-HCV antibodies (6.5%), patients with γ-GT levels ≥ 55 IU/L or history of alcoholism (8.5%), and patients with incomplete data (0.7%) were excluded.

### Data collection procedure

An interviewer-administered close-ended proforma was used to record age, sex, education, family income, cigarette smoking, co-illness, duration of diabetes, and family history of diabetes. Body weight (in kilograms) and height (in meters) were measured to calculate the BMI using the formula as follows: formula: BMI (Kg/m^2^) = [weight (in kilograms)] divided by [height (in meters)]^2^. Waist circumference (WC) in inches was measured to exclude central obesity, and abdominal ultrasonography (USG) was performed for diagnosing fatty liver. Random plasma glucose was estimated by glucose oxidase-phenol aminophenazone (GOD-PAP) method, hemoglobin A1c (HbA1c) by ion-exchange resin method, and liver enzymes by the International Federation of Clinical Chemistry (IFCC) method. The screening of hepatitis B and C infection was done by immuno-chromatographic technique (ICT). *PNPLA3* genotype was done by polymerase chain reaction (PCR), and restriction fragment length polymorphism (RFLP) method and *PNPLA3* allele frequencies were measured by Hardy-Weinberg equilibrium.

### PCR-RFLP

**Fig. 1:**
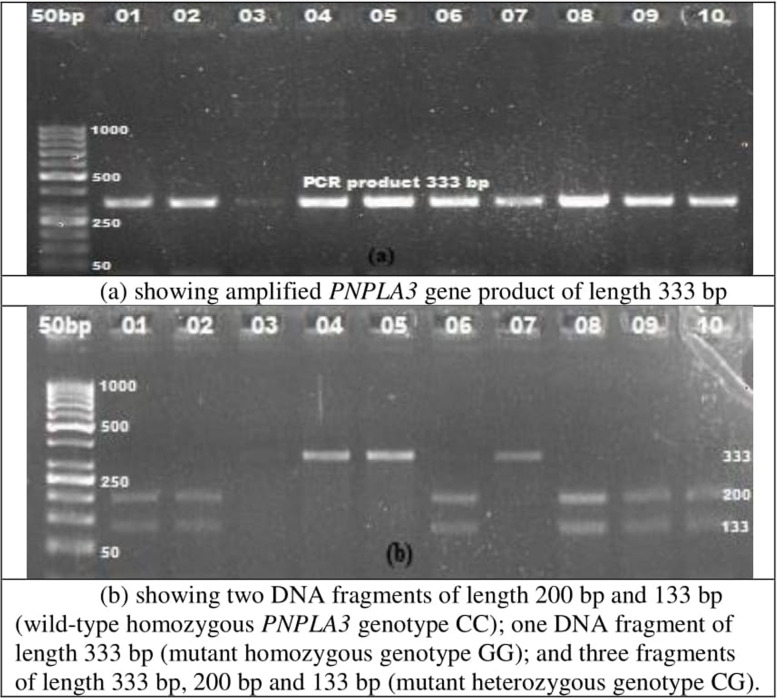
PCR-RFLP results of *PNPLA3* rs738409 polymorphism

The genomic deoxyribonucleic acid (DNA) was extracted by using the GeneJET Whole Blood Genomic DNA Purification Mini Kit. The amplification of *PNPLA3* gene was performed by PCR using the forward primer (5′-TGGGCCTGAAGTCCGAGGGT-3′) and the reverse primer (5′-CCGACACCAGTGCCCTGCAG-3′) as reported by Dutta (2011) [20]. The composition of PCR reaction mix and the optimized conditions are shown in Table 1. The restriction of amplified *PNPLA3* gene product (333 bp) was performed by using the BtsCI enzyme. The composition of the RFLP reaction mix is also shown in Table 1. The reaction mix was kept at 55 °C for overnight incubation. The digestion was stopped by keeping the reaction mix at 80 °C for 20 min. The restricted *PNPLA3* gene product was evaluated by 3.0% (w/v) agarose gel electrophoresis. Comparing with 50 bp DNA ladder, two DNA fragments of length 200 bp and 133 bp were labeled as *PNPLA3* genotype CC (wild-type homozygous), one DNA fragment of length 333 bp as genotype GG (mutant homozygous), and three fragments of length 333 bp, 200 bp, and 133 bp as genotype CG (mutant heterozygous) (Fig. 1).

**Table 1:** Composition of PCR-RFLP reaction mix and PCR conditions

***PCR reaction mix***		
Taq buffer (10×)		2.5 μL
MgCl_2_ (25 mM)		2.5 μL
dNTP mix (20 mM)		2.5 μL
Forward primer (10 μM)		1.5 μL
Reverse primer (10 μM)		1.5 μL
Taq DNA polymerase (5 units per μL)		0.5 μL
Water		12.0 μL
Genomic DNA		2.0 μL
Total volume		25.0 μL
***PCR conditions***		
Denaturation initial	94 °C for 2 min	01 cycle
Denaturation subsequent cycles each	94 °C for 30 s	35 cycles
Annealing	66 °C for 30 s	
Elongation initial	72 °C for 30 s	
Elongation final	72 °C for 5 min	01 cycle
***RFLP reaction mix***		
PCR product		10.0 μL
Nuclease-free water		18.0 μL
Tango buffer 10×		2.0 μL
BtsCI enzyme		1.0 μL
Total volume		31.0 μL

### Continuous variables

Each continuous variable was categorized into two groups to calculate the risk for NAFLD. Age categorized into ≤ 50 and > 50 years, family income into ≤ 20000 and > 20000 PKR, and duration of diabetes into < 10 and ≥ 10 years. Similarly, WC of male categorized into < 40 and ≥ 40 inch, WC of female into < 35 and ≥ 35 inch, BMI into < 30.0 and ≥ 30.0 Kg/m^2^, plasma glucose level into < 200 and ≥ 200 mg/dl, HbA1c level into ≤ 8.0 and ≥ 8.0 %, and alanine aminotransferase (ALT) level into < 40 and ≥ 40 IU/L.

### Statistical analysis

Statistical Package for Social Sciences (SPSS) version 26 was used for data analysis. Continuous variables were reported by using mean ± standard deviation and categorical variables by number (percent). *PNPLA3* genotype CC (wild-type homozygous), genotype GG (mutant homozygous), and genotype CG (mutant heterozygous) were categorized into dominant, recessive, and codominant model. The dominant model (GG + CG/CC) hypothesizes that the combination of mutant homozygous allele and mutant heterozygous allele can increase the risk of disease. The recessive model (GG/CG + CC) hypothesizes that mutant homozygous alleles can increase the risk of disease. The codominant models (GG/CC and CG/CC) hypothesize that mutant homozygous allele and mutant heterozygous allele can independently increase the risk of disease. The study population was categorized into NAFLD vs. non-NAFLD groups. Independent sample *t*-test and chi-square test were used to compare the means and frequencies between groups, respectively. Crosstabs analyses were performed to calculate the odds ratios (OR) with 95% confidence intervals for NAFLD. Then, the study population was categorized into obese-NAFLD, NAFLD alone, obese alone, and nonobese non-NAFLD groups. Binary and multinomial logistic regression analyses were performed under codominant, dominant, and recessive models. For each regression model, a total of 10 covariates were entered at step 1 with outcome variables obese NAFLD. The covariates were as follows: age, sex, income, education, smoking, comorbidity, duration of diabetes, family H/o diabetes, HbA1c level, and *PNPLA3* genotype. *p*-value ≤ 0.05 was considered as significant.

## Results

### Population characteristics

The participation of middle-aged adults (36–55 y) was the highest 64.3%, followed by older adults (≥ 56 y) 28.7% and young adults (18–35 y) 7.0%. The participation of females was twice higher than of males (66.7% vs. 33.3%). The respective means of family income and duration of diabetes were 25542 ± 18766 PKR/month and 6.8 ± 5.7 years. Among diabetics with any comorbidity (48.1%), the frequency of HTN was 38.8%, HTN + CVD 7.0% and CVD 2.3%. The frequency of central obesity was almost 3 times higher in females than in males (87.2% vs. 30.2%). The overall frequencies of overweight and obesity were 37.2% and 41.1%, respectively. Only 12.4% diabetics had good glycemic control (HbA1c < 7.0%). Overall, 52.7% diabetics were diagnosed with NAFLD, while 20.9% diabetics were carriers of *PNPLA3* genotype GG, 16.3% of CG, and 62.8% of CC. Consequently, the frequency of diabetics carrying mutant allele G was 29.05%. Other characteristics of the study population are shown in Table 2.

**Table 2:** Sociodemographic and clinical characteristics of study population (*n* = 129)

		***n*** (%)^**a**^	Mean ± SD^**b**^
**Age (years)**	**18–35 y (young)**	09 (7.0)	50.4 ± 11.5
	**36–55 y (middle-aged)**	83 (64.3)	
	**≥ 56 y (older)**	37 (28.7)	
**Sex**	**Female**	86 (66.7)	
**Education**	**Illiterate**	49 (38.0)	
**Family income (PKR/month)**			25542 ± 18766
**Duration of diabetes (years)**			6.7 ± 5.6
**Family history of diabetes**	**Yes**	93 (72.1)	
**Cigarette smoking**	**Yes**	06 (4.7)	
**Any comorbidity**	**Yes**	62 (48.1)	
**Waist circumference (inch)**^**c**^	**≥ 40 (male)**	13 (30.2)	38.1 ± 2.5
	**≥ 35 (female)**	75 (87.2)	39.2 ± 4.1
**Body mass index (Kg/m**^**2**^**)**	**25.0–29.9**	48 (37.2)	29.5 ± 5.6
	**≥ 30.0**	53 (41.1)	
**Random plasma glucose (mg/dl)**	**≥ 200**	90 (69.8)	238 ± 73
**HbA1c (%)**	**7.0–8.0**	38 (29.5)	9.0 ± 2.1
	**> 8.0**	75 (58.1)	
**Alanine transaminase (IU/L)**	**≥ 40**	17 (13.2)	32 ± 23
**Fatty liver**	**Yes**	68 (52.7)	
**PNPLA3 genotype**	**CC**	81 (62.8)	
	**CG**	21 (16.3)	
	**GG**	27 (20.9)	

^a^Table percent; ^b^overall means; ^c^sex-wise percentages and means; *PKR*, Pakistani rupee

### NAFLD in T2DM

Overall (*n* = 129), means of WC (39.5 ± 3.2 vs. 38.0 ± 3.9 inch; *p* = 0.022) and BMI (30.9 ± 5.8 vs. 28.1 ± 5.2 Kg/m^2^; *p* = 0.006) were significantly higher in NAFLD vs. non-NAFLD cases. In crosstabs analyses, females (*OR* = 2.998, 95% *CI* 1.398–3.430; *p* = 0.007), females with central obesity (*OR* = 5.333, 95% *CI* 1.301–21.869; *p* = 0.019), illiterates (*OR* = 3.067, 95% *CI* 1.446–6.505; *p* = 0.005), and obese (*OR* = 2.211, 95% *CI* 1.075–4.545; *p* = 0.046) showed significantly higher risk for NAFLD among overall diabetics. However, genotypes GG vs. CC (*OR* = 1.831, 95% *CI* 0.748–4.478; *p* = 0.266), CG vs. CC (*OR* = 1.436, 95% *CI* 0.545–3.780; *p* = 0.624), GG + CG vs. CC (*OR* = 1.644, 95% *CI* 0.797–3.391; *p* = 0.243), and GG vs. CG + CC (*OR* = 1.700, 95% *CI* 0.711–4.067; *p* = 0.326) had higher risk for NAFLD, but the association was not significant among overall diabetics (see Table 3).

**Table 3:** Factors associated with NAFLD in T2DM patients (*n* = 129)

Variable	***OR***^**a**^	95% CI for OR		***p***-value
		Lower	Upper	
**Age (≤ 50 years)**	1.459	0.721	2.953	0.382
**Sex (female)**	2.998	1.398	6.430	0.007
**Family income (≤ 20000 PKR/month)**	1.553	0.758	3.185	0.307
**Education (illiterate)**	3.067	1.446	6.505	0.005
**Smoking (yes)**	1.844	0.326	10.440	0.683
**Any comorbidity (yes)**	1.040	0.521	2.078	1.000
**Family history of diabetes (yes)**	0.853	0.394	1.849	0.837
**Duration of diabetes (≥ 10 years)**	1.700	0.711	4.067	0.326
**Waist circumference male (≥ 40 inch)**	2.000	0.523	7.647	0.324
**Waist circumference female (≥ 35 inch)**	5.333	1.301	21.869	0.019
**Body mass index (≥ 30.0 Kg/m**^**2**^**)**	2.211	1.075	4.545	0.046
**Alanine transaminase (≥ 40 IU/L)**	0.770	0.277	2.141	0.810
**Random plasma glucose (≥ 200 mg/dl)**	1.692	0.793	3.611	0.240
**HbA1c (> 8.0 %)**	1.559	0.771	3.151	0.289
**PNPLA3 genotype (GG/CC)**	1.831^b^	0.748	4.478	0.266
**PNPLA3 genotype (CG/CC)**	1.436^c^	0.545	3.780	0.624
**PNPLA3 genotype (GG + CG/CC)**	1.644	0.797	3.391	0.243
**PNPLA3 genotype (GG/CG + CC)**	1.700	0.711	4.067	0.326

^a^*n* = 129; ^b^*n* = 108; ^c^*n* = 102; *OR*, odds ratio; *CI*, confidence interval; *NAFLD*, nonalcoholic fatty liver disease; *T2DM*, type 2 diabetes mellitus. *p*-value ≤ 0.05 considered statistically significant

### NAFLD in obese T2DM

In binary logistic regression analyses, a total of 10 covariates were entered at step 1 with outcome variables obese NAFLD (*n* = 34) versus all others (*n* = 95). None out of ten covariates showed risk for NAFLD under codominant models; females (*AOR* = 4.010, 95% *CI* 1.156–13.912; *p* = 0.029) and *PNPLA3* genotype GG + CG (*AOR* = 3.303, 95% *CI* 1.099–9.920; *p* = 0.033) revealed significantly higher risk for NAFLD under dominant model, and illiterates (*AOR* = 3.506, 95% *CI* 1.080–11.375; *p* = 0.037) and *PNPLA3* genotype GG (*AOR* = 4.547, 95% *CI* 1.123–18.408; *p* = 0.034) revealed significantly higher risk for NAFLD under recessive model (see Table 4).

**Table 4:** Binary logistic regression for NAFLD in obese T2DM patients under dominant and recessive models (*n* = 129)

		2 × 2 crosstabs			Dominant model				Recessive model			
		NAFLD and BMI (≥ 30.0 Kg/m^**2**^)		***OR***	***AOR***	95.0% Cl for AOR		***p***-value	AOR	95.0% Cl for AOR		***p***-value
		Yes ***n*** (%)	No ***n*** (%)			Lower	Upper			Lower	Upper	
**Age (years)**	**≤ 50**	20 (26.3)	56 (73.7)	0.995	1.749	0.496	6.166	0.385	1.923	0.538	6.872	0.314
	**> 50**	14 (26.4)	39 (73.6)									
**Sex**	**Female**	28 (32.6)	58 (67.4)	2.977	4.010	1.156	13.912	0.029	3.208	0.934	11.015	0.064
	**Male**	06 (14.0)	37 (86.0)									
**Family income** **(PKR/month)**	**≤ 20000**	22 (27.2)	59 (72.8)	1.119	0.807	0.244	2.675	0.726	0.806	0.245	2.647	0.722
	**> 20000**	12 (25.0)	36 (75.0)									
**Education**	**Illiterate**	19 (38.8)	30 (61.2)	2.744	2.936	0.913	9.444	0.071	3.506	1.080	11.375	0.037
	**Literate**	15 (18.8)	65 (81.3)									
**Smoking**	**Yes**	0 (0.0)	06 (100.0)	0.000	0.000	0.000	.	0.999	0.000	0.000	.	0.999
	**No**	34 (27.6)	89 (72.4)									
**Any comorbidity**	**Yes**	18 (29.0)	44 (71.0)	1.304	1.616	0.530	4.924	0.399	1.779	0.572	5.536	0.320
	**No**	16 (23.9)	51 (76.1)									
**Family H/o diabetes**	**Yes**	26 (28.0)	67 (72.0)	1.358	1.798	0.497	6.504	0.371	2.148	0.590	7.820	0.246
	**No**	08 (22.2)	28 (77.8)									
**Duration of diabetes**	**> 10 years**	08 (29.6)	19 (70.4)	1.231	1.978	0.472	8.295	0.351	1.481	.341	6.434	0.600
	**≤ 10 years**	26 (25.5)	76 (74.5)									
**HbA1c (%)**	**> 8.0**	22 (29.3)	53 (70.7)	1.453	1.838	0.604	5.592	0.283	1.856	0.607	5.672	0.278
	**≤ 8.0**	12 (22.2)	42 (77.8)									
**PNPLA3 genotype**	**GG + CG**	19 (39.6)	29 (60.4)	2.883	3.303	1.099	9.920	0.033				
	**CC**	15 (18.5)	66 (81.5)									
**PNPLA3 genotype**	**GG**	12 (44.4)	15 (55.6)	2.909					4.547	1.123	18.408	0.034
	**CG + CC**	22 (21.6)	80 (78.4)									

*n* (%), row percent; *NAFLD*, nonalcoholic fatty liver disease; *T2DM*, type 2 diabetes mellitus; *BMI*, body mass index; *OR*, odds ratio; *AOR*, adjusted odds ratio; *CI*, confidence interval. *p*-value ≤ 0.05 considered statistically significant

In multinomial logistic regression analyses, a total of 10 covariates were entered at step 1 with outcome variables obese NAFLD (*n* = 34) versus obese alone (*n* = 19) or NAFLD alone (*n* = 34) or nonobese non-NAFLD (*n* = 42). In obese NAFLD versus obese alone and NAFLD alone, none out of ten covariates showed risk for NAFLD under any of three *PNPLA3* genotype models. Similarly, in obese NAFLD versus nonobese non-NAFLD, none showed risk for NAFLD under codominant model; however, females (*AOR* = 3.411, 95% *CI* 0.997–11.671; *p* = 0.051), and *PNPLA3* genotype GG + CG (*AOR* = 3.270, 95% *CI* 1.131–9.455; *p* = 0.029) revealed significantly higher risk for NAFLD under dominant model, and illiterates (*AOR* = 3.323, 95% *CI* 1.010–10.937; *p* = 0.048) and *PNPLA3* genotype GG (*AOR* = 4.534, 95% *CI* 1.221–16.826; *p* = 0.024) revealed significantly higher risk for NAFLD under recessive model (see Table 5).

**Table 5:** Multinomial regression analysis for NAFLD in obese T2DM patients under dominant and recessive models (*n* = 76)

		2 × 2 crosstabs			Dominant model				Recessive model			
		NAFLD and obese (BMI ≥ 30.0 Kg/m^**2**^)	Non-NAFLD and nonobese (BMI < 30.0 Kg/m^**2**^)	***OR***	***AOR***	95.0% Cl for AOR		***p***-value	***AOR***	95.0% Cl for AOR		***p***-value
		***n*** (%)	***n*** (%)			Lower	Upper			Lower	Upper	
**Age (years)**	**≤ 50**	20 (46.5)	23 (53.5)	1.180	1.671	0.493	5.668	0.410	1.562	0.464	5.257	0.471
	**> 50**	14 (42.4)	19 (57.6)									
**Sex**	**Female**	28 (58.3)	20 (41.7)	5.130	3.411	0.997	11.671	0.051	2.831	0.822	9.757	0.099
	**Male**	06 (21.4)	22 (78.6)									
**Family income** **(PKR/month)**	**≤ 20000**	22 (44.9)	27 (55.1)	1.020	0.786	0.255	2.424	0.675	0.653	0.209	2.042	0.464
	**> 20000**	12 (44.4)	15 (55.6)									
**Education**	**Illiterate**	19 (61.3)	12 (38.7)	3.170	2.626	0.818	8.429	0.105	3.323	1.010	10.937	0.048
	**Literate**	15 (33.3)	30 (66.7)									
**Smoking**	**Yes**	0 (0.0)	02 (100.0)	0.000	3.123 E-008	0.000	.^c^	0.998	3.998 E-008	0.000	.^c^	0.998
	**No**	34 (45.9)	40 (54.1)									
**Any comorbidity**	**Yes**	18 (51.4)	17 (48.6)	1.650	1.567	0.558	4.401	0.394	1.758	0.619	4.990	0.289
	**No**	16 (39.0)	25 (61.0)									
**Family H/o diabetes**	**Yes**	26 (48.1)	28 (51.9)	1.630	1.257	0.375	4.205	0.711	1.482	0.451	4.869	0.517
	**No**	08 (36.4)	14 (63.6)									
**Duration of diabetes**	**> 10 years**	08 (53.3)	07 (46.7)	1.540	1.614	0.378	6.885	0.518	1.205	0.276	5.267	0.804
	**≤ 10 years**	26 (42.6)	35 (57.4)									
**HbA1c (%)**	**> 8.0**	22 (51.2)	21 (48.8)	1.830	2.153	0.758	6.113	0.150	2.061	0.729	5.827	0.173
	**≤ 8.0**	12 (36.4)	21 (63.6)									
**PNPLA3 genotype**	**GG + CG**	19 (61.3)	12 (38.7)	3.170	3.270	1.131	9.455	0.029				
	**CC**	15 (33.3)	30 (66.7)									
**PNPLA3 genotype**	**GG**	12 (70.6)	05 (29.4)	4.040					4.534	1.221	16.826	0.024
	**CG + CC**	22 (37.3)	37 (62.7)									

*n* (%), row percent; *NAFLD*, nonalcoholic fatty liver disease; *T2DM*, type 2 diabetes mellitus; *BMI*, body mass index; *OR*, odds ratio; *AOR*, adjusted odds ratio; *CI*, confidence interval. *p*-value ≤ 0.05 considered statistically significant

## Discussion

NAFLD is a multisystem disease that not only results in progressive liver disease but also affects extrahepatic organs [21]. Various demographic [6–9], clinical [10], and genetic factors [11] showed association with the risk of NAFLD. Among genetic risk factors, *PNPLA3* rs738409 polymorphism demonstrated significant association with NAFLD. However, the characteristics of the study population varied across studies [17–19]. It is still unknown whether the association of *PNPLA3* rs738409 polymorphism with NAFLD exists or not among type 2 diabetic patients. Therefore, the present study aimed to evaluate the sociodemographic and genetic determinants of NAFLD in T2DM patients. The results showed that *PNPLA3* rs738409 polymorphism had higher risk for NAFLD under codominant, dominant, and recessive models, but was not associated with NAFLD in Pakistani adults with T2DM (all *p* > 0.05). It is in agreement with the results of Hsieh et al. (2015) who reported that rs738409 polymorphism had no association with NAFLD in Taiwanese patients with T2DM (*p* = 0.344) [16].

The prevalence of NAFLD is 50.0 to 70.0% in diabetic subjects [4]. An equivalent higher frequency of NAFLD 52.7% is obtained in the present study. Among sociodemographic factors, age, gender, obesity, and ethnicity are the most frequently reported risk factors for NAFLD. Hu et al. (2018) observed an increasing trend between advance age and occurrence of NAFLD (*OR* = 1.049; *p* = 0.607), but after adjustment, age had an inverse relation with NAFLD in adult Chinese (*OR* = 0.844; *p* = 0.157) [7]. Ferreira et al. (2010) also reported that age was not related with NAFLD in adults with T2DM (57.1 ± 10.9 vs. 57.6 ± 9.5 years; *p* = 0.818) [6]. In the same way, the present study found no significant relation between age and NAFLD (*OR* = 1.459; *p* = 0.382); however, an opposite trend was observed, where the highest frequency of NAFLD 57.1% was in age < 40 years and the lowest 42.9% in age > 60 years. Hu et al. (2018) reported that gender male was significantly associated with NAFLD in Chinese adults (*OR* = 3.484; *p* = < 0.001) [7]. Oppositely, Summart et al. (2017) reported that females had higher risk (*OR* = 1.3, 1.2–1.4) for NAFLD in Thai adults (> 40 years) [8]. Differently, Ferreira et al. (2010) reported that gender female was not associated with NAFLD in adults with T2DM (*p* = 0.939) [6], whereas gender female revealed significantly higher risk for NAFLD in the present study (*OR* = 2.998; *p* = 0.007). After adjustment, risk for NAFLD was further increased in obese females (*AOR* = 4.010; *p* = 0.029). Noteworthy, females with central obesity revealed significantly greater risk for NAFLD than of females without central obesity (*OR* = 5.333; *p* = 0.019). Education status also showed a significant relationship with the risk for NAFLD in the present study. Illiterates had significantly higher risk for NAFLD (*OR* = 3.067; *p* = 0.005); and after adjustment, risk for NAFLD was further increased in obese illiterates (*AOR* = 3.506; *p* = 0.037). Similar significant relation between low education level and FLD (*OR* = 0.704; *p* = 0.001) or NAFLD had been reported in other studies [22, 23]. The present study also showed association of elevated ALT levels with *PNPLA3* genotype GG/CC (*p* = 0.024), GG + CG/CC (*p* = 0.054), and GG/CG + CC (*p* = 0.018), which is consistent with other studies, where rs738409 polymorphism was associated with elevated AST levels (*p* = 0.039) [24] and ALT levels (*d* = 0.47) [17].

The human *PNPLA3* gene is expressed in various tissues of the body mainly in the liver. Its gene product, i.e., triacylglycerol lipase enzyme, mediates hydrolysis of TAG in adipocytes. However, *PNPLA3* rs738409 polymorphism causes loss of enzyme function resulting in the accumulation of TAG in the liver [12]. *PNPLA3* variants are the most common genetic risk factors leading to NAFLD in obese across different ethnic groups [25]. *PNPLA3* rs738409 variant had been reported as a significant risk factor for NAFLD in all genetic models [19] and in codominant model [17, 24, 26] among different study populations. However, the present study found no relationship between rs738409 polymorphism and risk of NAFLD in the whole T2DM group, which is in agreement with the findings of Hsieh et al. (2015) [16]. Differently, both binary and multinomial logistic regression analyses in the present study revealed that rs738409 polymorphism was significantly associated with NAFLD in obese diabetics, under dominant and recessive models (all *p* < 0.05).

## Conclusions

NAFLD and obesity were the most common comorbid diseases of T2DM in the setting. Gender female, being illiterate, and *PNPLA3* rs738409 polymorphism were significant risk factors of NAFLD among obese diabetic patients. Further research studies are needed to evaluate the association of *PNPLA3* rs738409 polymorphism and other genetic factors with the NAFLD particularly among obese diabetics.

## Data Availability

The data that support the findings of this study are available on request from the corresponding author. The data are not publicly available due to privacy or ethical restrictions.

## Abbreviations

ALT: Alanine aminotransferase
BMI: Body mass index
DNA: Deoxyribonucleic acid
FTO: Fat mass and obesity-associated
GCK: Glucokinase
GCKR: Glucokinase regulator
GOD-PAP: Glucose oxidase-phenol amino phenazone
GWAS: Genome-wide association studies
HbA1c: Hemoglobin A1c
HCC: Hepatocellular carcinoma
HBsAg: Hepatitis B surface antigen
HCV: Hepatitis C virus
HNF1A: Hepatocyte nuclear factor-1A
HTN: Hypertension
ICT: Immuno-chromatographic technique
IFCC: International Federation of Clinical Chemistry
LFC: Liver fat content
MBOAT7: Membrane-bound *O*-acyltransferase domain containing 7
MetS: Metabolic syndrome
NASH: Nonalcoholic steatohepatitis
NAFLD: Nonalcoholic fatty liver disease
OR: Odds ratios
PCR: Polymerase chain reaction
PNPLA3: Patatin-like phospholipase domain containing 3
RFLP: Restriction fragment length polymorphism
SPSS: Statistical Package for Social Sciences
T2DM: Type 2 diabetes mellitus
TAG: Triacylglycerol
TCF7L2: Transcription factor 7-like 2
TM6SF2: Transmembrane 6 superfamily member 2
USG: Ultrasonography
WC: Waist circumference
